# Comparative Analysis of Consumer Exposure to Resistant Bacteria through Chicken Meat Consumption in Germany

**DOI:** 10.3390/microorganisms9051045

**Published:** 2021-05-12

**Authors:** Carolina Plaza-Rodríguez, Octavio Mesa-Varona, Katja Alt, Mirjam Grobbel, Bernd-Alois Tenhagen, Annemarie Kaesbohrer

**Affiliations:** 1 Department Biological Safety, German Federal Institute for Risk Assessment (BfR), 10589 Berlin, Germany; Octavio.Mesa-Varona@bfr.bund.de (O.M.-V.); Katja.Alt@bfr.bund.de (K.A.); Mirjam.Grobbel@bfr.bund.de (M.G.); Bernd-Alois.Tenhagen@bfr.bund.de (B.-A.T.); Annemarie.Kaesbohrer@bfr.bund.de (A.K.); 2 Unit for Veterinary Public Health and Epidemiology, University of Veterinary Medicine, 1210 Vienna, Austria

**Keywords:** ESBL-/AmpC-producing *E. coli*, MRSA, model reusability, fskml standardized format, improper kitchen hygiene practices, household barbecue, cross-contamination, recontamination

## Abstract

Human exposure to bacteria carrying antimicrobial resistance (AMR) genes through the consumption of food of animal origin is a topic which has gained increasing attention in recent years. Bacterial transmission can be enhanced, particularly in situations in which the consumer pays less attention to hygiene practices, and consumer exposure to foodborne resistant bacteria through ready-to-eat foods could be increased. It has been demonstrated that even methicillin-resistant *Staphylococcus aureus* (MRSA) bacteria, which have low prevalence and concentration in raw chicken meat in Germany, may reach the consumer during barbecue events after failures in hygiene practices. This study aimed to quantify the consumer exposure to extended-spectrum beta-lactamase- (ESBL) or ampicillinase class C (AmpC) beta-lactamase-producing *E. coli* in Germany through the consumption of chicken meat and bread during household barbecues. The study considered cross-contamination and recontamination processes from raw chicken meat by using a previously-developed probabilistic consumer exposure model. In addition, a comparative analysis of consumer exposure was carried out between ESBL-/AmpC-producing *E. coli* and MRSA. Our results demonstrated that the probability of ESBL-/AmpC-producing *E. coli* reaching the consumer was 1.85 × 10^−5^ with the number of bacteria in the final serving averaging 332. Given the higher prevalence and concentration of ESBL-/AmpC-producing *E. coli* in raw chicken meat at retail compared to MRSA, comparative exposure assessment showed that the likelihood and extent of exposure were significantly higher for ESBL-/AmpC-producing *E. coli* than for MRSA. ESBL-/AmpC-producing *E. coli* was determined to be 7.6 times likelier (*p*-value < 0.01) than MRSA to reach the consumer, with five times the concentration of bacteria in the final serving (*p*-value < 0.01).

## 1. Introduction

The presence of bacteria carrying antimicrobial resistance (AMR) genes in foods of animal origin (as well as the possible role these foods fulfill as a source of human exposure to resistant bacteria through consumption) has gained increasing attention from researchers in recent years [1,2,3]. Direct contact between humans and livestock and indirect contact through the environment or contaminated meats have been described as the main routes of transmission of resistant bacteria between animals and humans [4,5,6]. Among these pathways, the relevance of meat as a possible source of human exposure to resistant bacteria has been widely discussed in the literature. The presence of genetically identical genes and plasmids carrying these genes in strains found in humans and meat samples [7,8] has been considered evidence of potential transmission of resistant bacteria through food. Resistant bacteria reaching the gastrointestinal tract through food consumption may constitute a public health risk, as antimicrobial-resistant genes may be transferred to pathogenic bacteria in the human intestine [9,10,11]. If these pathogens then cause an infection, it may lead to a decrease in the efficacy of the antibiotic treatments applied.

Consumer exposure to resistant bacteria can occur during handling of raw ingredients, e.g., through direct contact with the food handler’s hands, through consumption of undercooked food, or through consumption of food after cross-contamination or recontamination processes between different raw foods and ready-to-eat (RTE) foods. Transmission of resistant bacteria can happen at any point in the food production chain [12,13,14]. Indeed, the food processing industry applies regular hygiene controls in order to minimize the level of microbial contamination due to cross-contamination during processing [15]. Food preparation at home is not subject to any regulation and may be associated with more cross-contamination and recontamination processes, leading to unwanted consumer exposure [16,17,18,19]. Not washing hands after handling raw meat, putting cooked meat in the same dish as raw meat, or using the same knife or cutting board for raw meat and RTE foods are examples of practices that have been described as important cross-contamination and recontamination routes [17,20,21]. Moreover, these transmission pathways are even likelier to lead to bacterial exposure if the consumer pays less attention to hygiene measures. This may happen in festive events, such as household barbecues for families and friends, in which consumer exposure to foodborne resistant bacteria could be increased [16,22].

Our previous work characterized the possible consumer exposure to methicillin-resistant *Staphylococcus aureus* (MRSA) in Germany during a household barbecue (simulated through the consumption of a slice of bread and a piece of grilled chicken meat) [21]. For this purpose, a probabilistic model that estimated the probability and extent of MRSA transmission from raw chicken meat to the final serving through cross-contamination and recontamination was developed. In the contemplated scenario, MRSA could reach the consumer, but due to its low prevalence and concentration in raw chicken meat at retail in Germany, the probability and extent of consumer exposure were both estimated to be low. However, this might be different for extended-spectrum beta-lactamase- (ESBL) or ampicillinase class C (AmpC) beta-lactamase-producing *E. coli*. Although MRSA spreads more easily between surfaces and remains detectable in surfaces after a rinsing process [23], ESBL-/AmpC-producing *E. coli* have higher prevalence and concentration rates in raw chicken meat at retail in Germany [24,25,26,27,28].

ESBL-/AmpC-producing *E. coli* have been found in meat from all animal species across Europe [29,30]. However, despite the fact that the use of 3rd and 4th generation cephalosporins is not authorized for poultry in the European Union [31], the highest rates have been detected in broilers and chicken meat [29,32]. In Germany, the high prevalence and contamination levels of ESBL-/AmpC-producing *E. coli* in chicken meat at slaughterhouse and retail have been previously reported [27,28,32,33,34]. Consequently, it could be expected that a fraction of these bacteria are transferred from raw chicken meat to RTE foods through cross-contamination and recontamination processes—and ultimately, to the consumer through the intake of these contaminated foods.

The potential transmission of ESBL-/AmpC-producing *E. coli* from poultry to humans has prompted many investigations, including the development of probabilistic mathematical models that estimated the magnitude of transmission along the broiler production chain (including breeding farms, hatcheries, fattening farms, slaughterhouses and meat at retail) [13,35,36]. Some of these studies incorporated consumer exposure models that considered prevalence and concentration data of ESBL-/AmpC-producing *E. coli* or cephalosporin-resistant *E. coli* in raw chicken meat at retail in Belgium, Denmark or The Netherlands [13,18,37].

The objective of the present study was to quantify the probability and extent of human exposure to ESBL-/AmpC-producing *E. coli* through the consumption of a slice of bread and a portion of grilled chicken meat during a household barbecue in Germany. In addition, we performed a comparative analysis to (i) determine the differences between consumer exposure to ESBL-/AmpC-producing *E. coli* and MRSA through the consumption of chicken meat and bread within the contemplated scenario, and (ii) define which factors were the most relevant in relation to the final consumer exposure to foodborne resistant bacteria through chicken meat consumption.

## 2. Materials and Methods

### 2.1. Model Description

**Figure 1:**
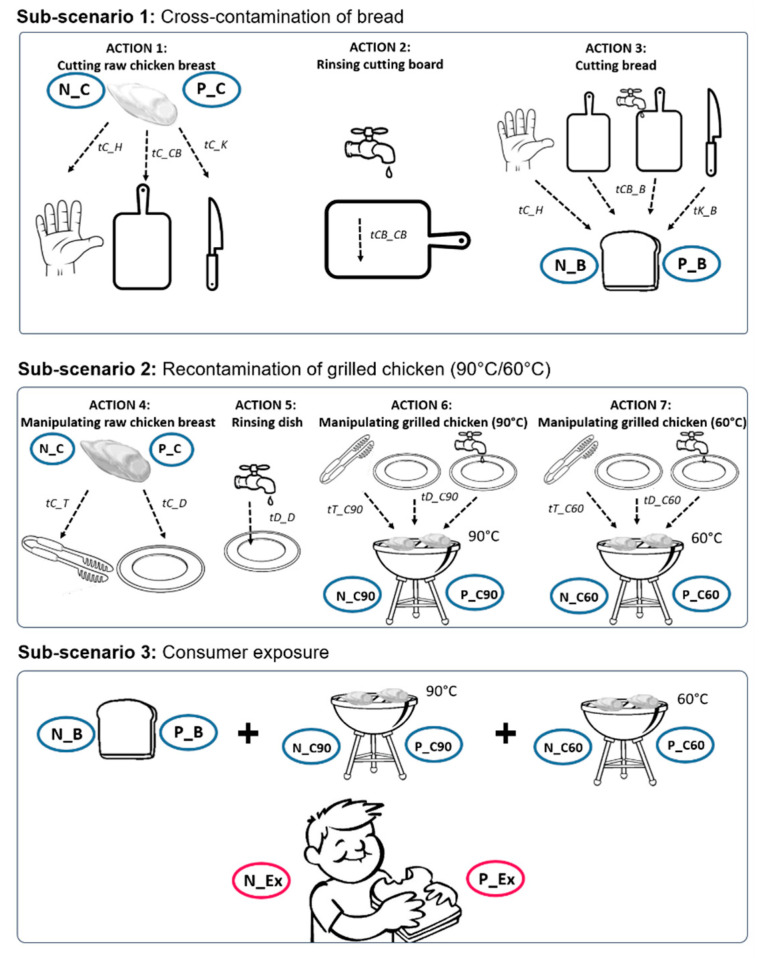
Detailed description of the contemplated scenario and sub-scenarios, including objects, transfer and persistence coefficients and probabilities of action occurrence. (tX_Y: transfer coefficient from X to Y; tX_X: persistence coefficient in X; N_X: Number of bacteria in X; P_X: Prevalence/Probability of bacteria in X; C: raw chicken meat; H: Hands; D: Dish; B: Bread; CB: Cutting board; K: Knife; T: Barbecue tong; C90: Grilled chicken at 90 °C; C60: Grilled chicken at 60 °C; pCF: probability of cutting the raw chicken first; pMF: probability that the raw chicken meat is manipulated first; pRCB: probability of rinsing the cutting board; pRD: probability of rinsing the dish; pC90: probability that the grilled chicken remains warm at 90 °C; pC60: probability of the grilled chicken to cool down to 60 °C).

The probabilistic model developed by Plaza-Rodriguez et al. (2019) [21] for the quantification of consumer exposure to MRSA was adapted in order to estimate the probability and extent of consumer exposure to ESBL-/AmpC-producing *E. coli*. This model described a specific scenario—possibly occurring during a household barbecue—in which grilled chicken was served and consumed together with a slice of bread. During the food preparation process, several actions were carried out and several objects were used, simulating failures in hygiene practices. These failures included raw chicken meat (contaminated with resistant bacteria) being handled and grilled and bread being cut into portions using the same objects that were used to manipulate the raw chicken meat. Depending on the routines and the hygienic practices applied, different transmission routes were generated between the raw chicken meat and the bread, and between the raw and the grilled chicken meat. Finally, the probability and extent of consumer exposure upon consumption of a portion of grilled chicken and bread was calculated. Figure 1 shows an overview of the main scenario and the sub-scenarios contemplated in the model, including the actions and objects involved and the transmission pathways generated between the objects. A more detailed description of the model, including a complete overview of the model equations, has been described by Plaza-Rodriguez et al. (2019) [21].

### 2.2. Model Parameterization

Model parameterization was mainly based on available data found in the literature or derived from the German Zoonoses Monitoring programs (Table 1).

It was assumed that initially only raw chicken meat was contaminated with ESBL-/AmpC-producing *E. coli*. Therefore, epidemiological data on the prevalence and concentration of ESBL-/AmpC-producing *E. coli* in chicken meat at the slaughterhouse or at retail in Germany were used. Prevalence data were collected from the German Zoonoses Monitoring programs carried out in 2013, 2016 and 2018 [24,25,26] and from the literature [32]. Data on the concentration of ESBL-/AmpC-producing *E. coli* in raw chicken meat at retail (CFU/g) were collected from the literature [27,28], including data obtained from samples collected at the slaughterhouse (from chicken fillets and neck skin samples used as meat surrogate). For those positive samples that presented ESBL-/AmpC-producing *E. coli* concentrations below the quantification limit, uniform distributions between the limit of detection (0.04 CFU/g) and the limit of quantification (Reich et al. (2016) [27]: 10 CFU/g (skin); von Tippelskirch et al. (2018) [28]: 150 CFU/g (skin) and 15 CFU/g (filet)) were applied.

Prevalence and concentration data were fitted to theoretical distributions using the R package fitdistrplus [42], evaluating the goodness of fitting by using the Kolmogorov–Smirnov test and visual analysis.

Transfer and persistence coefficients between food and kitchen objects were adapted from the cross-contamination and recontamination experiments carried out by Fetsch (2015) [23] for ESBL-/AmpC-producing *E. coli*. Transfer coefficients that showed values greater than 100 in this study were assumed to be 100%. In addition, in the original work, no bacteria were observed after rinsing the cutting board and the dish or after handling the hot meat at 90 °C. However, as we wanted to consider the worst-case scenario, it was assumed that these receiving surfaces could be contaminated with a number of bacteria below the limit of detection. For this reason, it was assumed for these cases that there was a transmission of 100 CFU per square centimeter, and such transmission ranges were calculated accordingly.

In order to be able to perform a comparative analysis between ESBL-/AmpC-produ-cing *E. coli* and MRSA, the same technical assumptions and values used by Plaza-Rodriguez et al. (2019) [21] for other parameters (like the surface of the objects involved or the probabilities of occurrence of the different routines performed during food preparation) were used.

### 2.3. Model Simulation

The original FSK-ML formatted model from Plaza-Rodriguez et al. (2019) [21], was downloaded from the public repository in the compress file format. fskx (https://zenodo.org/record/3240621#.XP1CyIgzbIU, last accessed on 11 May 2021). It contained all relevant files needed for model reusability [43,44,45].

The R model script and the visualization script were then imported into R 3.6.3 on a Windows 10 platform and adapted accordingly. Monte Carlo simulations with 100,000 iterations were applied in order to simulate many repetitions of the serving preparation. Then probability distributions were calculated for the probabilities of one CFU of ESBL-/AmpC-producing *E. coli* to be transferred from raw chicken meat and the number of CFU transferred to bread, grilled chicken and the final serving as a result of cross-contamination and recontamination processes. In order to visualize the simulated probability distributions, frequency histograms were represented alongside boxplots. Boxplots were used to display the distribution of data based on median, interquartile range (IQR) (from first quartile (Q1) to third quartile (Q3)), minimum (Q1 − 1.5 × IQR) and maximum (Q3 + 1.5 × IQR). The values of the outliers were also included in the boxplot.

As performed by Depoorter et al. (2012) [13], the probability of consumer exposure to four different exposure doses (10, 100, 1000 and 10,000 CFU/serving) was estimated within the contemplated scenario.

In addition, to better understand the impact of hygiene practices on final consumer exposure, different what-if scenarios were elaborated. To accomplish these, the model was executed, varying the default numerical values established for the probabilities of the occurrence of the different hygiene practices from 0 to 100%. The what-if scenarios contemplated variations in (i) the probability of the raw chicken meat being cut before the bread; (ii) the probability of the raw chicken meat being manipulated before the grilled chicken meat; (iii) the probability of the cutting board being rinsed after cutting the raw chicken meat; (iv) the probability of the dish being rinsed after being used for raw chicken meat; (v) the probability of the grilled chicken meat remaining warm at 90 °C or (vi) cooling to 60 °C at the time of manipulation before consumption; and (vii) the prevalence of ESBL-/AmpC-producing *E. coli* in raw chicken meat at retail.

### 2.4. Comparative Analysis of Consumer Exposure between ESBL-/AmpC-producing E. coli and MRSA by Using the Same Probabilistic Model

In order to evaluate the differences in consumer exposure to different resistant bacteria within the contemplated scenario, a comparative analysis of consumer exposure was carried out between ESBL-/AmpC-producing *E. coli* and MRSA. For this, the model deve-loped by Plaza-Rodriguez et al. (2019) [21] for MRSA was executed again, and the probability distributions obtained as output were compared with the output of the current study for ESBL-/AmpC-producing *E. coli*.

The normality of the probability distributions obtained for both microorganisms were initially evaluated by using the Kolmogorov–Smirnov test and visual analysis. Normality was not observed in any of the cases, and therefore a nonparametric test was applied to calculate the differences between both microorganisms. In addition, as the number of observations in the two groups was quite large and based on the central limit theorem [46], a parametric test was applied.

An initial descriptive comparison was carried out. Probability distributions obtained for both microorganisms (for the probability of transmission and the number of resistant bacteria transferred to bread, grilled chicken meat and the final serving) were graphically compared. To that end, we used density histograms (including the mean value), violin plots (that included the kernel density plot and the boxplot) and interval plots (including the mean and 95% confidence intervals). Subsequently, the parametric T-Test and the non-parametric Wilcoxson test were applied to determine whether the differences observed visually and analytically in the mean and the median values between the two microorganisms were statistically significant.

### 2.5. Model Reusability and Exchange

The model presented in this work was converted into the standardized data format called Food Safety Knowledge Markup Language (FSK-ML) using the software tool FSK-Lab [43] and shared in a public model repository (https://zenodo.org/record/4748645, last accessed on 11 May 2021) in order to provide transparency to the mo-deling generation process and to facilitate its further use.

## 3. Results

### 3.1. Estimation of Consumer Exposure to ESBL-/AmpC-producing E. coli

**Figure 2:**
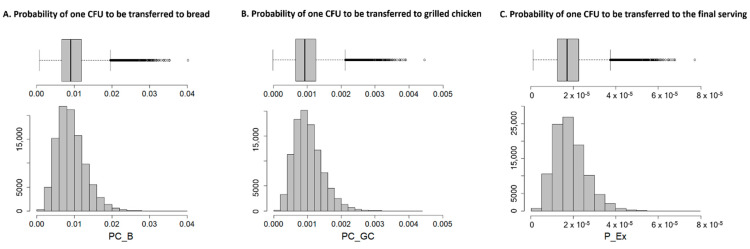
Estimated relative frequencies on the probabilities of one CFU of ESBL-/AmpC-producing *E. coli* transferring to (**A**) bread (PC_B), (**B**) grilled chicken (PC_GC) and (**C**) the final serving (P_Ex), due to the cross-contamination and recontamination processes contemplated in the model.

Figure 2 provides a graphical description of the simulated probability distributions of one cell to be transferred from raw chicken meat to bread (PC_B) and grilled chicken meat (PC_GC) and therefore to the final serving (P_Ex), which takes into account the pre-valence of ESBL-/AmpC-producing *E. coli* in chicken meat at retail in Germany. The average probability of cross-contamination between raw chicken meat and bread was slightly higher (9.18 × 10^−3^) than the probability of recontamination of grilled chicken (1.02 × 10^−3^) (Table 2). Considering the probabilities of occurrence of both processes, in 95% of simulations the probability of one cell reaching the consumer through consumption of the final serving during a barbecue event was lower than 3.25 × 10^−5^.

**Figure 3:**
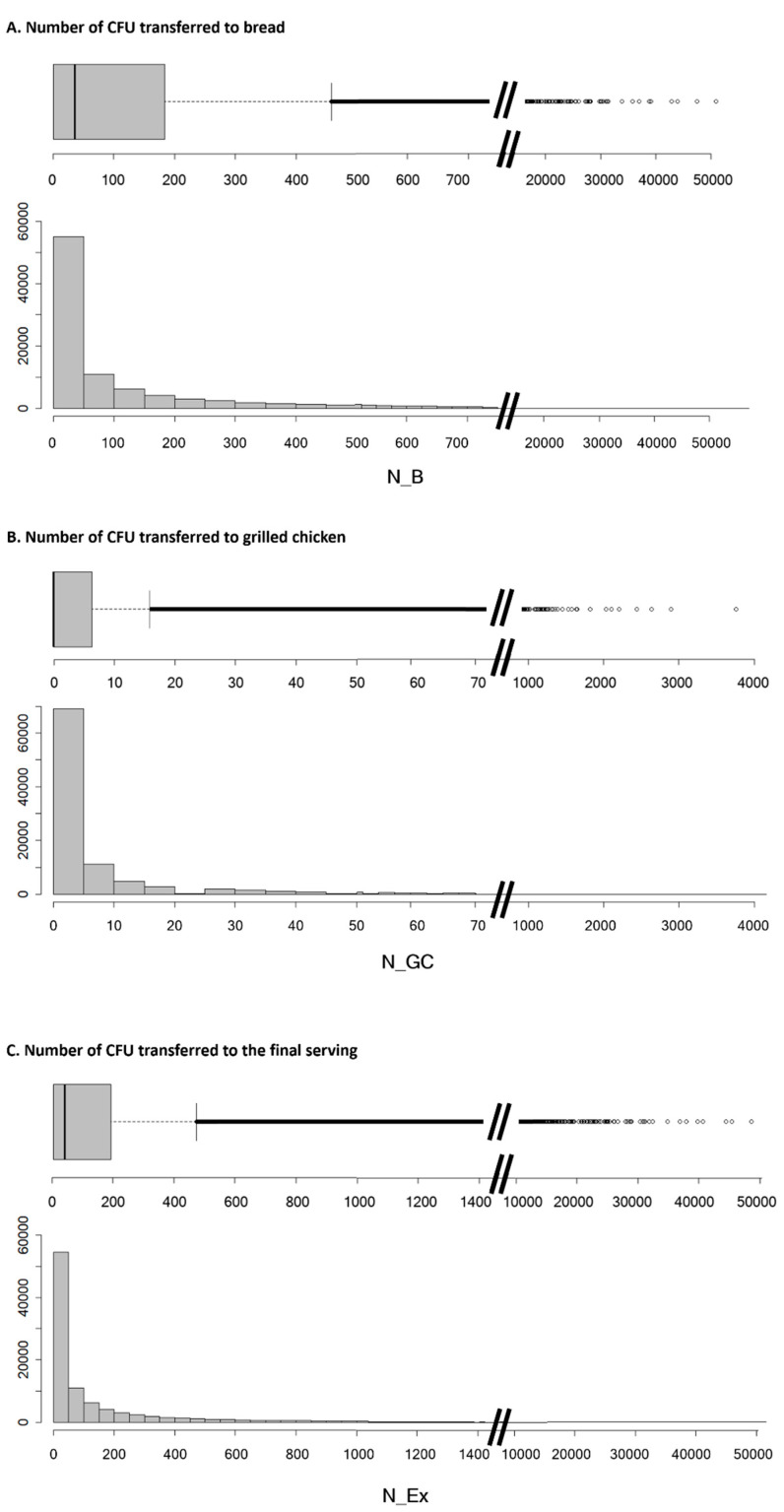
Estimated relative frequencies on the number of ESBL-/AmpC-producing *E. coli* transferred from raw chicken meat to (**A**) bread (N_B), (**B**) grilled chicken (N_GC) and (**C**) the final serving (N_Ex) due to the cross-contamination and recontamination events contemplated in the model.

The number of cells that would be transferred from contaminated raw chicken meat to bread (N_B) and grilled chicken meat (N_GC)—and therefore to the final serving (N_Ex)—through the different surfaces contemplated in this scenario is presented in Figure 3. The average number of bacteria to which the final consumer would be exposed was estimated to be 332 CFU per serving. Most of this contamination was attributed to cross-contamination of the bread (319 CFU/serving) and not to recontamination of the grilled chicken (which only contributed to a limited extent (13 CFU/serving)). It should be noted that there was high variability and dispersion in the simulated number of transferred bacteria. Fifty percent of the simulations estimated that a transmission of less than 36 cells would occur for the bread and the final serving, with no transmission for grilled chicken meat. However, there were many outliers, which were far away from the interquartile range and the whiskers represented in the boxplot. These outliers represented around 14% of all simulations and showed values that went up to more than 5000 CFU/serving for the grilled chicken and 119,000 for the bread and the final serving.

**Figure 4:**
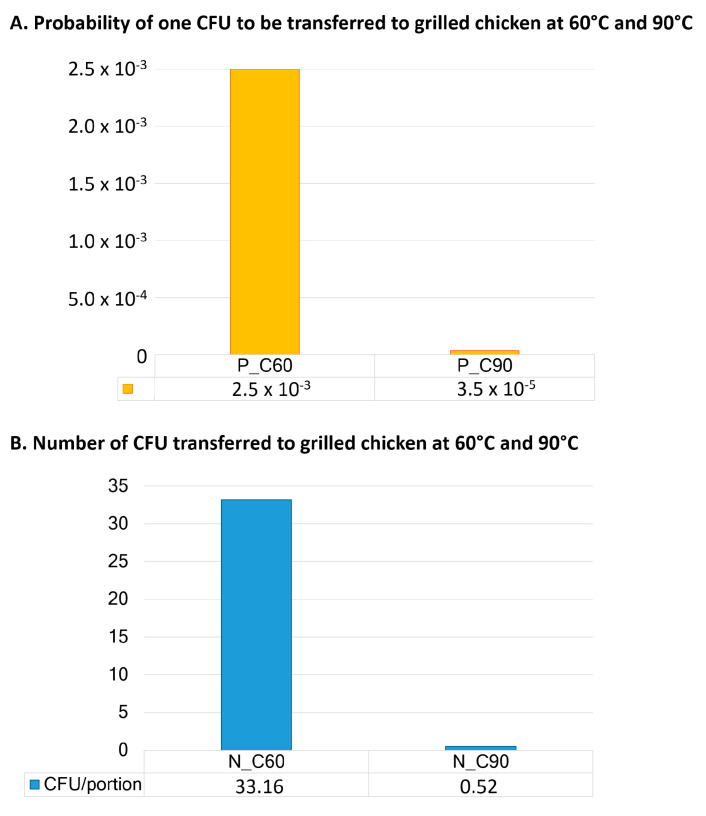
Influence of the temperature of the grilled chicken at time of manipulation before consumption (90 °C/60 °C) on the (**A**) mean probability of one CFU of ESBL-/AmpC-producing *E. coli* being transferred to grilled chicken and (**B**) number of ESBL-/AmpC-producing *E. coli* transferred (on average) to a portion of grilled chicken.

When we compared the two scenarios set out in the model in relation to the tempe-rature of the grilled meat at the time of manipulation before consumption, we noticed large differences in the probability and extent of bacterial transmission (Figure 4). In cases in which the grilled chicken remained hot (at 90 °C), the probability of recontamination of the grilled chicken was 3.5 × 10^−5^ on average. If the meat cooled down to 60 °C this probability increased to 2.5 × 10^−3^. At 90 °C, no bacterial transmission occurred in 97.2% of si-mulations. However, at 60 °C, around 33 CFU/portion was transferred on average, and only 69.2% of simulations resulted in no transmission.

In cases in which the raw chicken meat was contaminated, the average probabilities of consumer exposure to more than 10, 100, 1000 and 10,000 CFU of ESBL-/AmpC-producing *E. coli* in a portion of grilled chicken meat and a slice of bread handled under the simulated situation were 62.1%, 34.6%, 7.2% and 0.3%, respectively (Table 3).

### 3.2. Impact of Hygiene Practices in Consumer Exposure to ESBL-/AmpC-producing E. coli (What-if Scenarios)

**Figure 5:**
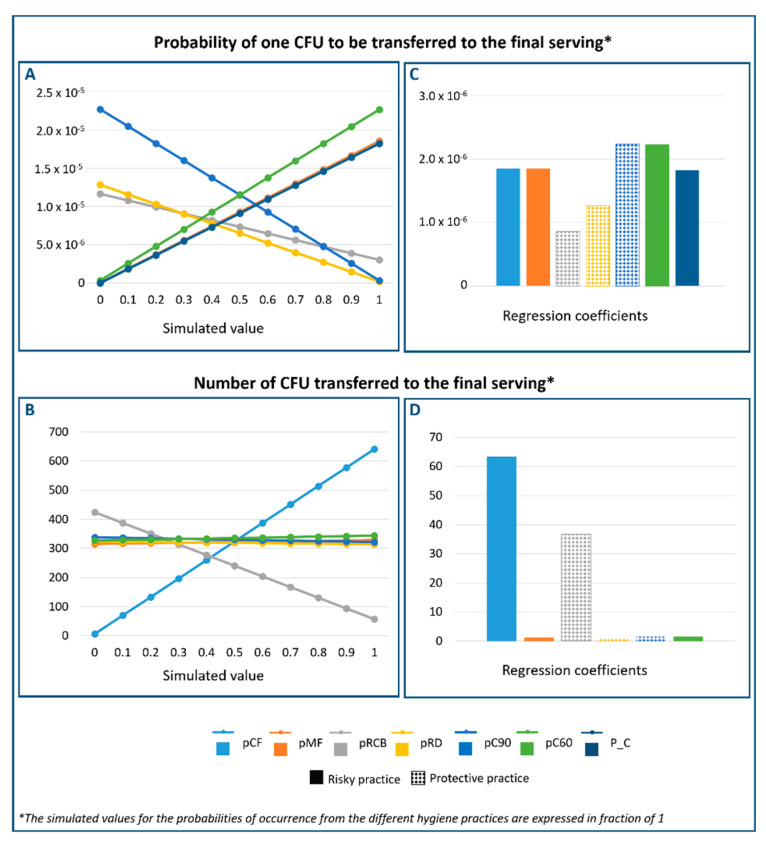
(**A**) Probability of one CFU of ESBL-/AmpC-producing *E. coli* being transferred and (**B**) the number of CFU transferred to the final serving as a function of the hygiene practices. Regression coefficients between the probabilities of action occurrence and the probability of one CFU being transferred to the final serving (**C**), and the number of CFU transferred to the final serving (**D**), including their protective or risky effect on consumer exposure. (i) probability that the raw chicken meat is cut before cutting the bread (pCF); (ii) probability that the raw chicken meat is manipulated before the grilled chicken meat (pMF); (iii) probability that the cutting board is rinsed after cutting the raw chicken meat and before cutting the bread (pRCB); (iv) probability that the dish is rinsed after being used for raw chicken meat (pRD); probability that the grilled chicken remains warm (pC90); (v) prevalence of ESBL-/AmpC-producing *E. coli* in chicken meat at retail (P_C)).

The impact of hygiene practices during food preparation on the consumer exposure to ESBL-/AmpC-producing *E. coli* is graphically presented in Figure 5A,B. In addition, the values of the regression coefficients, which demonstrate the magnitude of their effect, are presented in Figure 5C,D.

As can be seen from Figure 5A,C, three of the evaluated hygiene practices demonstrated a protective effect on the probability of consumer exposure: (i) the probability of handling the chicken at 90 °C (pC90), (ii) the probability of rinsing the cutting board before cutting the bread (pRCB) and (iii) the probability of rinsing the dish before placing the grilled chicken (pRD). In other words, an increase in the probability of performing these practices would reduce the probability of consumer exposure. On the other hand, the prevalence of ESBL-/AmpC-producing *E. coli* at retail (P_C), the probability of cutting the raw chicken meat before the bread (pCF), the probability of handling the raw chicken meat before the grilled chicken (pMF) and the probability of handling the grilled chicken that has been cooled to 60 °C (pC60) all increased the probability of ESBL-/AmpC-producing *E. coli* being transferred to the final serving. Among these practices, those related with the temperature of the chicken meat at the time of manipulation after cooking and before consumption exerted the greatest influence on the probability of consumer exposure.

Regarding the number of bacteria transmitted to the final serving in cases in which the raw chicken meat was contaminated, most of the applied hygiene practices did not show a great impact. Only the probability of rinsing the cutting board before cutting the bread (pRCB) demonstrated a protective effect. The probability of cutting the chicken before the bread (pCF) increased the number of cells that reached the final serving. For example, increasing the probability of cutting the raw chicken meat before the bread from 50% to 100% was associated with an increase of 300 CFU in the final serving. On the other hand, increasing the percentage of rinsing the cutting board from 30 to 100% decreased the number of CFU in the final serving by more than 200 cells.

### 3.3. Comparative Analysis of Consumer Exposure to ESBL-/AmpC-producing E. coli and MRSA

**Figure 6:**
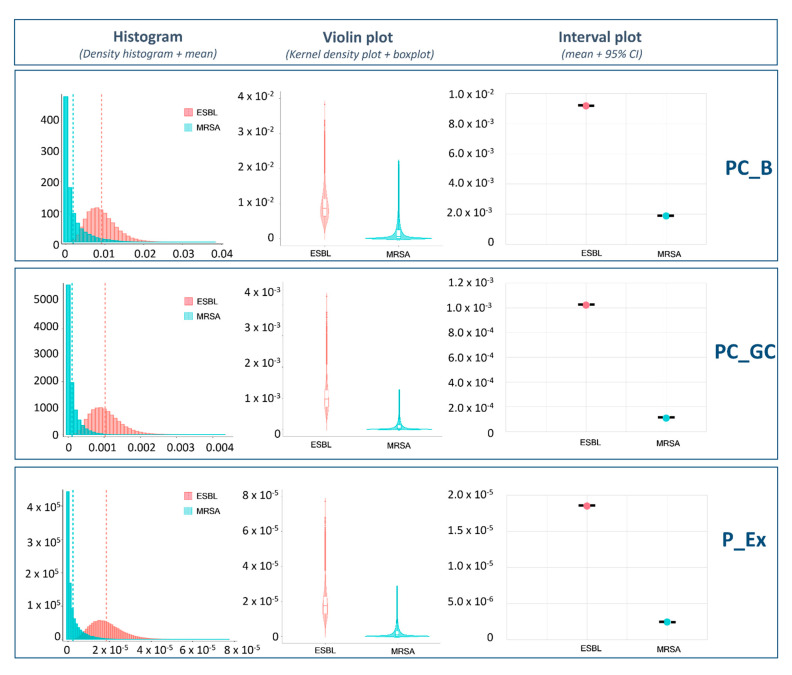
Comparative analysis of consumer exposure between ESBL-/AmpC-producing *E. coli* and MRSA, where the probability of one CFU to be transferred to bread (PC_B), grilled chicken (PC_GC) and the final serving (P_Ex) is graphically represented.

Figure 6 compares the probabilities of one cell being transferred from raw chicken meat to bread (PC_B), grilled chicken meat (PC_GC) and the final serving (P_Ex) for MRSA and ESBL-/AmpC-producing *E. coli*. There were clear differences between the bacterial species. Most of the MRSA simulations indicated that the probability of bacterial transmission was equal to zero. In contrast, simulations for ESBL-/AmpC-producing *E. coli* led to a broader range of values. Comparing the mean values, the average probability of consumer exposure to ESBL-/AmpC-producing *E. coli* was 7.6 times higher than the probability for MRSA exposure through consumption of the final serving.

**Figure 7:**
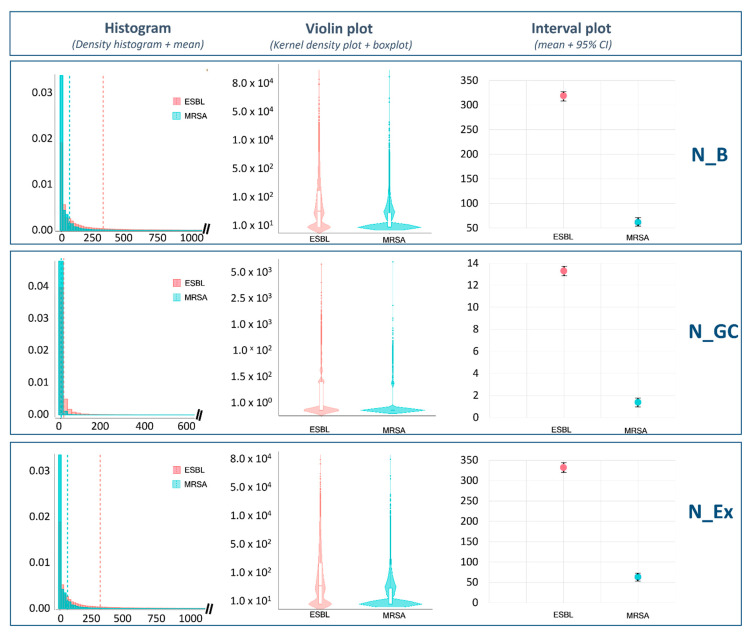
Comparative analysis of consumer exposure between ESBL-/AmpC-producing *E. coli* and MRSA where the number of CFU transferred to bread (N_B), grilled chicken (N_GC) and the final serving (N_Ex) is graphically represented.

Figure 7 shows the number of bacteria that would be transmitted from raw chicken meat to bread (N_B), to grilled chicken (N_GC) and to the final serving (N_Ex), in cases where the raw meat was contaminated with ESBL-/AmpC-producing *E. coli* or MRSA. There was a large overlap between the density histograms of ESBL-/AmpC-producing *E. coli* and MRSA. However, the violin plots showed that MRSA had wider sections in the areas close to zero. That means that more simulations indicated a transmission of very few cells to the final serving. In contrast, at this level, the sections in the violin plots were not that wide for ESBL-/AmpC-producing *E. coli* and the outcomes of the simulations were more widely distributed. All this supposes that 5 times more cells of ESBL-/AmpC-producing *E. coli* than MRSA cells would be found in the final serving in cases in which the raw chicken meat was contaminated.

Table 4 shows the analytical and statistical comparisons of the mean and the median values between the model outputs from both microorganisms. In all cases, the differences observed between ESBL-/AmpC-producing *E. coli* and MRSA were statistically significant. Therefore, the probability and extent of consumer exposure to ESBL-/AmpC-produ-cing *E. coli* was significantly higher than the probability and extent of consumer exposure to MRSA in the contemplated scenario.

## 4. Discussion

The present study provides a quantitative probabilistic estimation of consumer exposure to ESBL-/AmpC-producing *E. coli* through the consumption of grilled chicken meat and bread, probably contaminated from raw chicken meat during a household barbecue. In addition, a comparative analysis of consumer exposure between two types of resistant bacteria present in raw chicken meat was conducted. For that purpose, we made use of a model previously created and shared in a publicly available model repository. Currently, it is not general practice to systematically provide the model equation in a standardized and reusable way when a study is published within the food microbiology domain [47]. This has made previously developed models published in scientific journals difficult to reuse. The fact that this model was shared in a standardized format (FSK-ML) [43,44] increased the reliability of the model and minimized efforts during the model generation process. It also facilitated the comparison between the outputs of this study and the previous study [45,47]. The model used is a probabilistic model that describes the different cross-contamination and recontamination pathways that can lead to the transmission of resistant bacteria from contaminated raw chicken meat to RTE foods (in our case, grilled chicken and bread). It allowed investigation of the possible impact of different hygiene practices on the probability and extent of consumer exposure to resistant bacteria. To be able to reuse the model, we had to adapt it to the specific case of ESBL-/AmpC-producing *E. coli*. For this, specific parameters were used, including the prevalence and concentration of ESBL-/AmpC-producing *E. coli* in chicken meat at retail and the transmission and persistence coefficients between different surfaces. The findings and conclusions presented in this paper were based on specific scenarios, data and assumptions—which should be taken into account. These factors may have resulted in overestimation or underestimation of consumer exposure probabilities. Gathering further data—for example, on the probability of carrying out certain hygienic practices during a barbecue event—could contribute to more accurate consumer exposure estimates.

The results obtained in this study further support the hypothesis that chicken meat could be one of the major contributors to human exposure to ESBL-/AmpC-producing *E. coli* through food consumption [7,37,48]. With the current prevalence of ESBL-/AmpC-producing *E. coli* in chicken meat at retail, the estimated probability of ESBL-/AmpC-producing *E. coli* reaching consumers is low. However, if the chicken meat is contaminated with this resistant bacterium, the average number of bacteria in the final serving may be remarkably high. This is of particular concern, since our model assumed that the consumer would only eat one serving. Even higher probabilities and levels of exposure could be expected if the consumer were to eat more than one serving, or if other RTE foods (like raw vegetable sticks or fresh salads) were consumed after also being contaminated from raw chicken meat contact.

It should also be noted that the estimated number of bacteria transferred to bread and grilled chicken varied widely from none or a few cells to thousands of cells, if the raw meat was contaminated. Although in most cases bacteria would neither reach the bread nor the grilled chicken, the number of bacteria that could be transmitted to the final serving reached 10^5^ CFU in some simulations. To the best of our knowledge, the concentration of ESBL-/AmpC-producing *E. coli* in chicken meat at retail has not been extensively studied in Germany. Only two studies provided quantitative estimations at the slaughterhouse [27,28]. Both studies analyzed neck skin samples and used them as a meat surrogate. Only one of them conducted quantitative analyses on chicken fillets. Even though ESBL-/AmpC-producing *E. coli* concentration in neck skin was slightly higher than in fillets [28], we decided to include data from both origins, since chicken meat is often sold with skin and we wanted to consider the worst-case scenario for our model. Furthermore, in the cited studies, a great variability in quantitative estimations was observed. Although most of the samples were not contaminated (or the contamination level of ESBL-/AmpC-producing *E. coli* was below the limit of quantification), contamination in some samples exceeded 10^5^ CFU/g. As we wanted to represent the worst-case scenario, we decided not to correct any data entry from the selected bibliography in order to calculate the probabi-lity distribution on the concentration of ESBL-/AmpC-producing *E. coli* at retail. Hence, the great variability observed in our predictions mirrored the variability found in the original studies that were used as the starting point of our model.

Current knowledge on the impact ESBL-/AmpC-producing *E. coli* ingestion via food consumption on human health is still insufficient [9]. Although it has been suggested that the consumption of chicken meat could be related to the acquisition of multidrug-resistant *E. coli,* causing urinary tract infections [49], in most cases, human infections with ESBL-/AmpC-producing *E. coli* are preceded by asymptomatic carriage [50]. In broilers, it has been demonstrated that a dose of 10^1^ CFU/mL is capable of causing colonization with a subsequent excretion >10^6^ CFU/g in feces 72 h after inoculation [51]. In humans, quantitative information is not available; however, there is evidence that even low levels of exposure may lead to colonization, with a probability of colonization correlated with the level of exposure [52]. This colonization in the long term/after exposure to additional risk factors may lead to infections, and could also suppose a later excretion and a risk of household transmission between humans [52,53]. In addition, it has been demonstrated in vitro that resistant bacteria reaching the gastrointestinal tract can cause a horizontal plasmid-based resistance gene transfer to other commensal or pathogenic bacteria in the human intestine [9,10,11]. Since consumers do not know the degree of contamination of the raw chicken meat that they manipulate and cook at home, the risk of cross-contamination and recontamination of the final serving with ESBL-/AmpC-producing *E. coli* cells should not be overlooked.

Previous studies estimated the consumer exposure to ESBL-/AmpC-producing *E. coli* and 3rd generation cephalosporin resistant *E. coli* through the ingestion of chicken meat [13,18,37] using prevalence and concentration data from other countries like Belgium, Denmark and The Netherlands. Evers et al. (2017) [18] estimated that 1.73 cells of ESBL-/AmpC-producing *E. coli* would be found in a contaminated portion of chicken meat in The Netherlands. The probability of a portion being contaminated was estimated to be 6.85 × 10^−2^. In comparing these results with ours, we noted that the probability of exposure was higher than that calculated by our model, and yet the number of transmitted cells was lower. When calculating the average number of cells per serving, taking into account the percentage of contaminated rations, our model estimated that, on average, 5.8 × 10^−3^ cells would be in every serving (versus 1.20 × 10^−1^ estimated by Evers et al. (2017)) [18].

Depoorter et al. (2012) [13] calculated the probability of the consumer being exposed to four arbitrarily chosen consumer exposure doses of 3rd generation cephalosporin resistant *E. coli* (10, 100, 1000 and 10,000 CFU/meal), using data from Belgium. We reproduced this approach with our model in order to be able to compare the results. Depoorter et al. (2012) [13] estimated that the probability of the consumer being exposed to more than 1000 CFU of 3rd generation cephalosporin resistant *E. coli* during the consumption of a meal containing broiler meat was about 1.5%. Our model, however, calculated this probability to be 7.19%, a probability nearly five times higher.

These discrepancies observed between our results and those from previous models may be due to differences in the assumptions and scenarios contemplated during the design and parameterization of the models. Depoorter et al. (2012) [13] calculated the pre-valence and concentration of bacteria in the raw meat in previous modules of the model, instead of making use of the real prevalence and concentration data of chicken meat at retail. Depoorter et al. (2012) [13] and Evers et al. (2017) [18] used transmission and persistence coefficients adapted from other bacteria such as *Salmonella spp*. rather than specific coefficients for ESBL-/AmpC-producing *E. coli* (as we did). They considered consumer exposure through cross-contamination or undercooked processes, but they did not include the recontamination of cooked chicken and used vegetables instead of bread as RTE food. All these factors—and possibly others not listed herein—make direct comparisons between outputs difficult. Therefore, any interpretation of the differences found has to be made with caution.

Depoorter et al. (2012) [13] stated that cross-contamination was more important than insufficient heating when considering consumer exposure to ESBL-/AmpC-producing *E. coli* via meat. Our model did not include the effect of insufficient heating with the integration of predictive models of bacterial thermal inactivation. It assumed that all contamination was superficial and that bacteria were completely inactivated during the cooking process. Therefore, the effect of temperature contemplated in our model referred only to the temperature of the meat when handling it after cooking and before consumption. Our results demonstrated that, when evaluating the effect of temperature on the presence of ESBL-/AmpC-producing *E. coli* in the final serving, it is important to consider the inactivation of bacteria that are present in raw chicken meat, and also of those that could reach the cooked meat through recontamination processes once the cooking process has fi-nished. In other words, the recontamination of grilled chicken after the cooking process depended on the temperature on the surface of the meat when it was manipulated with unwashed/contaminated kitchen utensils. In our scenario, this was represented by mani-pulation with barbecue tongs and by placing the grilled chicken on the dish previously used for the raw chicken meat. The higher the temperature on the surface of the meat when handling it with objects that have been in contact with raw chicken, the smaller the probability of bacteria reaching the consumer, because these bacteria will be inactivated by the temperature on the surface of the meat. If the surface temperature of the meat is lower, these bacteria might survive [23], and could reach the consumer. In fact, our study revealed that the probability of handling the grilled chicken meat at 90 °C (versus doing so after it has cooled to 60 °C) had the greatest impact on the probability of consumer exposure to ESBL-/AmpC-producing *E. coli*.

However, most of the contamination of the final serving was attributable to the cross-contamination process from raw chicken meat to bread; recontamination of the grilled chicken played a minor role. In our model, the probability of rinsing the cutting board before cutting the bread, and the probability of cutting the raw chicken meat before the bread exerted the greatest effect on the number of cells that reached the final serving if the raw chicken meat was contaminated. Therefore, basic hygienic measures in the kitchen, such as cutting the bread before the raw chicken meat, using different utensils or rinsing them after cutting the raw chicken meat and before cutting the bread, considerably reduced the number of ESBL-/AmpC-producing *E. coli* bacteria that reached the final consumer.

Comparing our current results with those obtained previously for MRSA [21], we noted that the impact of the hygiene routines in the bacterial count was similar. However, there were clear differences with respect to the influence of those practices on the probability of consumer exposure. For MRSA, the prevalence at retail had a major impact, while hygienic routines did not have great influence. These differences could be explained by the fact that the prevalence of ESBL-/AmpC-producing *E. coli* at retail is much greater than the prevalence of MRSA. Therefore, the higher the prevalence, the greater the effect of hygienic routines on the probability and extent of consumer exposure. Based on that, we concluded that, if the prevalence was low, the effect of hygienic measures on the probability of exposure would likewise be low, in comparison with the effect of changes in pre-valence. However, if the prevalence was high, strict adherence to hygienic measures could have a greater effect on the likelihood of consumer exposure than minor changes in pre-valence.

Under the scenario contemplated in this study, our results showed that ESBL-/AmpC-producing *E. coli* as more likely to reach the consumer through the consumption of a portion of grilled chicken meat and a slice of bread during a barbecue in Germany than MRSA. Furthermore, if the raw chicken meat was contaminated, the amount of bacteria reaching the final consumer was significantly higher for ESBL-/AmpC-producing *E. coli* than for MRSA. As one of our purposes was to compare MRSA and ESBL-/AmpC-producing *E. coli* under the same scenario, the same technical assumptions and values used by Plaza-Rodriguez et al. (2019) [21] for other parameters (e.g., the surface of the objects involved or the probabilities of occurrence of the different routines performed during food preparation) were used. Therefore, the key differences between the two models were the prevalence and bacterial concentration data on raw chicken meat at retail in Germany—and in the transfer and persistence coefficients of both bacteria.

The transfer and persistence coefficients were adapted from Fetsch (2015) [23]. In that study, significant differences between the transfer and persistence coefficients of ESBL-/AmpC-producing *E. coli* and MRSA from the different surfaces involved in the cooking process were identified. MRSA was transferred more effectively between surfaces in most of the scenarios studied and remained detectable after rinsing the cutting board and the dish, in contrast to ESBL-/AmpC-producing *E. coli*. The author attributed these differences to the diversity in the origin and the properties of the bacteria. MRSA is mucosal and a skin colonizer, and ESBL-/AmpC-producing *E. coli* are intestinal bacteria [54]. Our model showed that, although MRSA has higher transmission and persistence rates, the probability and extent of consumer exposure to ESBL-/AmpC-producing *E. coli* was statistically higher because of the high prevalence and concentration of ESBL-/AmpC-producing *E. coli* in chicken meat. This underlines that the most important factor in determining the probability and extent of consumer exposure to resistant bacteria is not restricted to the transfer rates, the type of bacteria or their ability to adhere to surfaces. In fact, the prevalence and concentration of those bacteria in raw meat is very significant. Hence, the implementation of reduction policies for antibiotic use in primary production and compliance with good manufacturing/hygiene practices in slaughterhouses and processing plants are essential to reduce the prevalence and contamination level of resistant bacteria in raw meat at retail, therefore minimizing consumer exposure to resistant bacteria through food consumption.

## 5. Conclusions

Although the risk of consumer exposure to ESBL-/AmpC-producing *E. coli* through consumption of grilled chicken meat and bread possibly contaminated from raw chicken meat appears to be small, our results showed that a considerable number of bacteria would reach the final serving if the raw chicken meat was contaminated. The probability and extent of consumer exposure to ESBL-/AmpC-producing *E. coli* are significantly higher than those of MRSA—although MRSA can be transferred between surfaces more efficiently than ESBL-/AmpC-producing *E. coli*. Therefore, the implementation of a drug reduction policy in primary production and compliance with good manufacturing/hygiene practices in slaughterhouses, cutting plants and during food processing are essential to minimize consumer exposure to resistant bacteria. Additionally, strict adherence to hygienic measures during household food manipulation is of particular importance in those cases where the prevalence of resistant bacteria is high in raw chicken meat at retail. Therefore, handling raw meat properly, using different kitchen utensils for raw and RTE food and washing surfaces between different steps of food preparation would help to reduce, or even eliminate, the risk of bacterial exposure through contaminated food.

## Figures and Tables

**Table 1:** Detailed description of the model parameters including definitions, default numerical values and sources of information (ESBL = ESBL-/AmpC-producing *E. coli*).

Sub-Scenario	Parameter Type	Notation	Parameter	Unit *	Description	Parameter Reference
1–2	Prevalence and bacterial concentration in raw chicken meat at retail	P_C	Prevalence of ESBL (P) in raw chicken meat (C) at retail		rgamma(5.87, 11.56)	[24,25,26,32]
		N_C	Contamination level (N) on raw chicken meat (C) at retail	CFU/cm^2^	rweibull(0.33, 62.89)/1.25	[27,28]
1	Transfer coefficients and bacterial persistence after rinsing	tC_H	Transfer coefficient (t) from raw chicken meat (C) to hand (H)		0.0384	[23]
		tC_CB	Transfer coefficient (t) from raw chicken meat (C) to cutting board (CB)		0.0342	[23]
		tC_K	Transfer coefficient (t) from raw chicken meat (C) to knife (K)		0.0103	[23]
		tH_B	Transfer coefficient (t) from hands (H) to bread (B)		0.0300	[38]
		tCB_B	Transfer coefficient (t) from cutting board (CB) to bread (B)		1	[23]
		tK_B	Transfer coefficient (t) from knife (K) to bread (B)		1	[23]
		tCB_CB	Persistence coefficient of ESBL in cutting board (CB) after rinsing		0.0134	[23]
	Probabilities of action occurrence	pCF	Probability (p) that the raw chicken meat (C) is cut first (F) (before cutting the bread)		0.50	[39]
		pRCB	Probability (p) that the cutting board (CB) is rinsed (R) after cutting the raw chicken meat and before cutting the bread		0.28	[40]
		pCB	Probability (p) that the cutting board (CB) is not rinsed after cutting the raw chicken meat and before cutting the bread		1-pRCB	[21]
	Surfaces involved	SB_H	Bread contaminated surface (SB) from hand (H)	cm^2^	90	[41]
		SB_CB	Bread contaminated surface (SB) from cutting board (CB)	cm^2^	runif (63, 80)	[41]
		SB_K	Bread contaminated surface (SB) from knife (K)	cm^2^	19.60	[41]
2	Transfer coefficients and bacterial persistence after rinsing	tC_D	Transfer coefficient (t) from raw chicken meat (C) to dish (D)		0.018	[23]
		tC_T	Transfer coefficient (t) from raw chicken meat (C) to barbecue tong (T)		0.0089	[23]
		tD_C90	Transfer coefficient (t) from dish (D) to grilled chicken that remains at 90 °C (C90)		0.0027	[23]
		tT_C90	Transfer coefficient (t) from barbecue tong (T) to grilled chicken that remains at 90 °C (C90)		0.0038	[23]
		tD_C60	Transfer coefficient (t) from dish (D) to grilled chicken that remains at 60 °C (C60)		0.3774	[23]
		tT_C60	Transfer coefficient (t) from barbecue tong (T) to grilled chicken that remains at 60 °C (C60)		0.0038	[23]
		tD_D	Persistence coefficient of ESBL in dish (D) after rinsing		0.0027	[23]
	Probability of action occurrence	pMF	Probability (p) that the raw chicken meat is manipulated (M) first (F) (before grilled chicken is manipulated)		1	[21]
		pC90	Probability (p) that the grilled chicken remains warm (C90) when is manipulated		0.60	[21]
		pC60	Probability (p) that the grilled chicken cools to 60 °C (C60) before being manipulated		1-pC90	[21]
		pRD	Probability that the dish (D) is rinsed(R) after being used for raw chicken meat manipulation		0.28	[40]
		pD	Probability (p) that the dish (D) is not rinsed after being used for raw chicken meat		1-pRD	[21]
	Surfaces involved	SGC_D	Grilled chicken contaminated surface (SGC) from dish (D)	cm^2^	22.14	[41]
		SGC_T	Grilled chicken contaminated surface (SGC) from barbecue tong (T)	cm^2^	14.17	[41]

* Transfer/persistence coefficients, probabilities and prevalence values are expressed as a fraction of 1.

**Table 2:** Model estimates on the probabilities of one CFU of ESBL-/AmpC-producing *E. coli* transferring from raw chicken meat to bread (PC_B), grilled chicken (PC_GC) and final serving (P_Ex), and the number of CFU transferred from raw chicken meat to bread (N_B), grilled chicken (N_GC) and final serving (N_Ex).

		Minimum	Q1	Median	Mean	SD	Q3	Maximum
**Probability of one CFU being transferred from raw chicken meat** ***(expressed as a fraction of 1)***	PC_B	7.40 × 10^−^^4^	6.44 × 10^−^^3^	8.68 × 10^−^^3^	9.18 × 10^−^^3^	3.77 × 10^−^^3^	1.14 × 10^−^^2^	3.83 × 10^−^^2^
	PC_GC	8.24 × 10^−5^	7.17 × 10^−^^4^	9.66 × 10^−^^4^	1.02 × 10^−^^3^	4.20 × 10^−^^4^	1.27 × 10^−^^3^	4.27 × 10^−^^3^
	P_E×	1.49 × 10^−^^6^	1.30 × 10^−^^5^	1.75 × 10^−^^5^	1.85 × 10^−^^5^	7.59 × 10^−^^6^	2.29 × 10^−^^5^	7.72 × 10^−^^5^
**Number of CFU transferred from raw chicken meat** ***(CFU/serving)***	N_B	0	0	35	319	1301	183	114,201
	N_GC	0	0	0	13	60	6	5618
	N_Ex	0	0	37	332	1360	189	119,820

**Table 3:** Probability of the consumer being exposed to specific ESBL-/AmpC-producing *E. coli* concentrations through consumption of a portion of bread, grilled chicken and the final serving.

Exposure Dose	>10 CFU/g	>100 CFU/g	>1000 CFU/g	>10,000 CFU/g
Bread	61.8%	34.1%	6.9%	0.3%
Grilled chicken	19.8%	2.9%	0.1%	0.0%
Final serving	62.1%	34.6%	7.2%	0.3%

**Table 4:** Comparative analysis of consumer exposure between methicillin-resistant Staphylococcus aureus and ESBL-/AmpC-producing *E. coli.*

	ESBL-/AmpC-producing *E. coli*			Methicillin-Resistant Staphylococcus aureus			Diff. Means	*p*-Value *(ParametricT-Test)*		*p*-Value *(Non-Parametric Wilcoxon Test)*
	Mean	95% CI	Median	Mean	95% CI	Median			Diff. Medians	
**PC_B**	9.18 × 10^−3^	(9.16 × 10^−3^–9.20 × 10^−3^)	8.68 × 10^−3^	1.88 × 10^−3^	(1.86 × 10^−3^–1.90 × 10^−3^)	5.78 × 10^−4^	7.30 × 10^−3^	<0.001	8.10 × 10^−3^	<0.001
**PC_GC**	1.02 × 10^−3^	(1.02 × 10^−3^–1.02 × 10^−3^)	9.66 × 10^−4^	1.07 × 10^−4^	(1.06 × 10^−4^–1.08 × 10^−4^)	3.29 × 10^−5^	9.15 × 10^−4^	<0.001	9.33 × 10^−4^	<0.001
**P_Ex**	1.85 × 10^−5^	(1.84 × 10^−5^–1.85 × 10^−3^)	1.75 × 10^−5^	2.44 × 10^−6^	(2.42 × 10^−6^–2.47 × 10^−6^)	7.50 × 10^−6^	1.60 × 10^−5^	<0.001	9.98 × 10^−6^	<0.001
**N_B**	318.91	(310.85–326.97)	35.07	61.84	(56.15–67.53)	0.00	257.07	<0.001	35.07	<0.001
**N_GC**	13.30	(12.93–13.68)	0.00	1.38	(1.24–1.51)	0.00	11.92	<0.001	0.00	<0.001
**N_Ex**	332.21	(323.79–340.64)	36.63	63.22	(57.39–69.05)	0.00	268.99	<0.001	36.63	<0.001

## Data Availability

The model script generated during the current study is available in the Zenodo repository at (https://zenodo.org/record/4748645, last accessed on 11 May 2021).
